# Was the fruit in front of me or near the river? Biological relevance and reference frame preferences in object-location memory across the lifespan

**DOI:** 10.3758/s13423-026-02961-0

**Published:** 2026-07-17

**Authors:** Silvia Serino, Giuseppe Riva, Claudia Repetto, Karine Marie Goulene, Marco Stramba-Badiale, Cosimo Tuena

**Affiliations:** 1 Department of Psychology, Università degli Studi Milan–Bicocca, Piazza Dell’Ateneo Nuovo, 1 20126, Milan, Italy; 2 Applied Technology for Neuro-Psychology Lab, IRCCS Istituto Auxologico Italiano, Milan, Italy; 3 Humane Technology Lab, Università Cattolica del Sacro Cuore, Milan, Italy; 4 Department of Psychology, Università Cattolica del Sacro Cuore, Milan, Italy; 5 Department of Medicine, Neurology and Rehabilitation, IRCCS Istituto Auxologico Italiano, Milan, Italy; 6 Department of Theoretical and Applied Sciences, Research, Center in Applied Psychology (CePsi), eCampus University, Novedrate, Italy

**Keywords:** Allocentric, Egocentric, Spatial cognition, Aging, Adaptive memory

## Abstract

Spatial memory research has traditionally focused on how environmental features shape the use of egocentric and allocentric reference frames, with little attention to the role of the objects themselves. Here, using two datasets collected with a virtual reality object-location memory task, we investigated whether the biological relevance of objects modulates spatial memory performance depending on the reference frame used at retrieval. Participants encoded the positions of biological entities and artifacts and retrieved them using either egocentric or allocentric recall cues. In Study 1, young adults (*N* = 37) showed a significant interaction between object category and recall cue: biological entities were recalled more accurately under allocentric than egocentric retrieval, while artifacts showed the opposite pattern, though the category difference was significant only under allocentric retrieval. In Study 2, older adults and patients with amnestic mild cognitive impairment (*N* = 80) showed a preserved biological advantage in overall spatial performance, whereas the interaction with recall cue was no longer observed, a pattern consistent with the reduced allocentric contribution typically associated with aging. These findings provide initial evidence that object categories may shape the retrieval of spatial locations.

## Introduction

For our ancestors, remembering the location of food sources or potential threats played a central role in survival. Similarly, today we need to remember where we left our keys, locate the car in a parking lot, or find the way back to that lovely café. These scenarios underline the importance of remembering not just *where* something is, but *what* is located there. Remembering where objects are located—memory for object locations—is essential for orienting ourselves, planning routes from origin to goal, and ultimately navigating our surroundings effectively (Llana et al., 2025; Manns & Eichenbaum, 2009; Postma & De Haan, 1996).

While extensive research has investigated how environmental features, such as boundaries and landmarks, shape spatial memory (Buckley et al., 2021; Bullens et al., 2010; Doeller et al., 2008; Sheynikhovich et al., 2025), considerably less attention has been devoted to how the object’s features may influence spatial memory for its location. Object locations can be encoded within two spatial reference frames (Klatzky, 1998): egocentric and allocentric. Egocentric representations code and maintain spatial position relative to the observer (e.g., “*the fruit tree is behind me*”); they require continuous updating during movement and are particularly efficient for immediate actions in the environment. Allocentric representations, on the other hand, code and maintain objects’ spatial position in a framework that is centered on elements external to the observer (e.g., “*the fruit tree is near the river*”), and coherently support flexible retrieval of spatial information from any location within the environment (Burgess, 2006; Ekstrom & Hill, 2023).

These two spatial representations rely on partially distinct neural circuits: egocentric processing primarily engages the posterior parietal cortex (Stein, 1992), with the dorsal striatum storing stimulus–response associations for navigational choices (Chersi & Burgess, 2015; Doeller et al., 2008). Allocentric processing depends mainly on the hippocampus, where place cells encode specific locations (Ekstrom et al., 2003; O’Keefe & Dostrovsky, 1971), and on connected medial temporal lobe structures: the entorhinal cortex, which provides metric information through grid cells (Buzsáki & Moser, 2013), and the parahippocampal cortex, which supports scene recognition (Epstein et al., 2007). Critically, allocentric processing is particularly vulnerable to aging (Ladyka‐Wojcik & Barense, 2021): physiological aging is associated with a preferential decline in allocentric spatial processing, with relative preservation of egocentric abilities (Colombo et al., 2017; Lester et al., 2017), a pattern that worsens in amnestic mild cognitive impairment (aMCI; Tuena et al., 2021) and in dementia due to Alzheimer’s Disease (AD; Serino et al., 2025). These deficits reflect the early vulnerability of hippocampal and parahippocampal structures to AD neuropathology (Long & Holtzman, 2019).

Existing evidence suggests that spatial memory can be modulated by the salience of what is associated with a given location. In rodents, fear conditioning produces place cell reorganization that stabilizes over time, even when the physical environment remains unchanged (Moita et al., 2004; Wang et al., 2012), and reward locations are encoded by a dedicated pool of hippocampal cells that maintains consistent goal representations across different environments (Gauthier & Tank, 2018). In humans, emotional arousal enhances memory for the spatial position of pictures (Mather & Nesmith, 2008), and aesthetic liking specifically improves egocentric memory for artwork locations in virtual environments (Babo-Rebelo et al., 2022). The biological relevance of an object may play a similar, and perhaps more crucial, role.

A robust finding in the memory literature is that living things are remembered better than nonliving ones, a phenomenon known as the animacy effect (for a recent meta-analysis, see Cheng et al., 2025). This effect has been attributed to the greater attentional capture by animate stimuli (New et al., 2007), to greater semantic feature overlap among living items (Xiao et al., 2016), and, critically, from an evolutionary perspective, to the adaptive value of detecting and retaining conspecifics, animals, and foods relevant for survival (Nairne & Pandeirada, 2016; Nairne et al., 2017). This memory advantage has only recently been extended to the spatial domain: spatial and temporal details of encoding are better remembered for animate than inanimate items (Gelin et al., 2018), and participants appear to be more accurate at retrieving the spatial positions of animate than inanimate objects (Lhoste et al., 2025).

Importantly, from the perspective of adaptive memory, what matters for survival extends beyond animate entities: forgetting where a fruit tree was located can directly affect survival, while the position of a rock is less critical (Nairne, 2014). Thus, here we adopt a broader framework that distinguishes biological entities (animals, plants, fruits) from artifacts (manufactured objects). Encoding and retaining the location of biological elements in a stable, observer-independent format, namely within an allocentric reference frame, would support flexible retrieval of that information even after the observer has moved through the environment. Artifacts, which are typically interacted with through immediate, body-directed action, may instead be more easily maintained in transient, egocentric reference frames. Whether the biological relevance of objects also influences the spatial reference frame in which their location is represented remains unexplored.

Moreover, from a neurobiological standpoint, living and nonliving things partially engage distinct regions along the lateral–medial axis of the ventral visual stream (Konkle & Caramazza, 2013; Sha et al., 2015). This organization extends into the medial temporal lobe, where object representations in the perirhinal cortex, parahippocampal cortex, and hippocampus are shaped by animacy, with distinct organizational patterns across these brain structures (Blumenthal et al., 2018). Together, these converging lines of evidence raise the possibility that biological and artificial objects, may be differentially accessible depending on the spatial reference frame used at retrieval. In addition, if the spatial recall of biological entities benefits preferentially from allocentric processing, then the progressive allocentric decline characteristic of aging should selectively affect the spatial memory of biological objects.

The current work tested this prediction using data from two studies (Tuena et al., 2025, 2026) that employed a comparable virtual reality object–location memory task from previous research (Guderian et al., 2015; Tuena et al., 2025), in which spatial recall is tested with both egocentric and allocentric retrieval conditions. Crucially, in both original studies, the object sets included equal numbers of biological entities and artifacts, allowing a balanced test of category effects. In Study 1, we tested whether object category modulates spatial reference frame use in healthy young adults (YA). Based on the adaptive memory framework (Nairne & Pandeirada, 2016; Nairne et al., 2017), we predict an interaction: spatial recall error should be lower for biological objects under allocentric retrieval and for artifacts under egocentric retrieval. In Study 2, we extended this investigation to cognitively healthy older adults (OA) and patients with aMCI, to determine whether the allocentric decline that characterizes pathological aging alters the relationship between object category and spatial reference frame use. Because biological items are expected to benefit from allocentric retrieval, while allocentric processing declines in aging and is further compromised in aMCI (Colombo et al., 2017; Tuena et al., 2021), we predict a weaker interaction: specifically, the interaction between object category and spatial reference frame should be attenuated, or even abolished, in older groups.

## Methods

### Participants

In Study 1, 37 YA (18–30 years) were recruited. In Study 2, participants included 40 OA and 40 individuals diagnosed with aMCI, with all participants being 65 years of age or older. Study 1 was approved by the Ethics Committee of the Catholic University of Milan (code: 67–21); participants were recruited at psychology courses at the Catholic University of Milan. Study 2 was conducted in accordance with the Declaration of Helsinki and approved by the Ethics Committee of Istituto Auxologico Italiano (code: 2023_01_31_11); participants were recruited at the Outpatient Clinic of the Department of Medicine, Neurology and Rehabilitation, IRCCS Istituto Auxologico Italiano–Mosè Bianchi, Milan. Written informed consent was obtained from the participants before they participated in the studies. For details of the inclusion and exclusion criteria, see the original publications (Study 1: Tuena et al., 2025; Study 2: Tuena et al., 2026). The present analyses were conducted on previously collected datasets; hence, sample sizes were originally determined to test hypotheses about spatial frame processing and were not specifically powered to detect category-related effects.

### Materials and apparatus

**Fig. 1:**
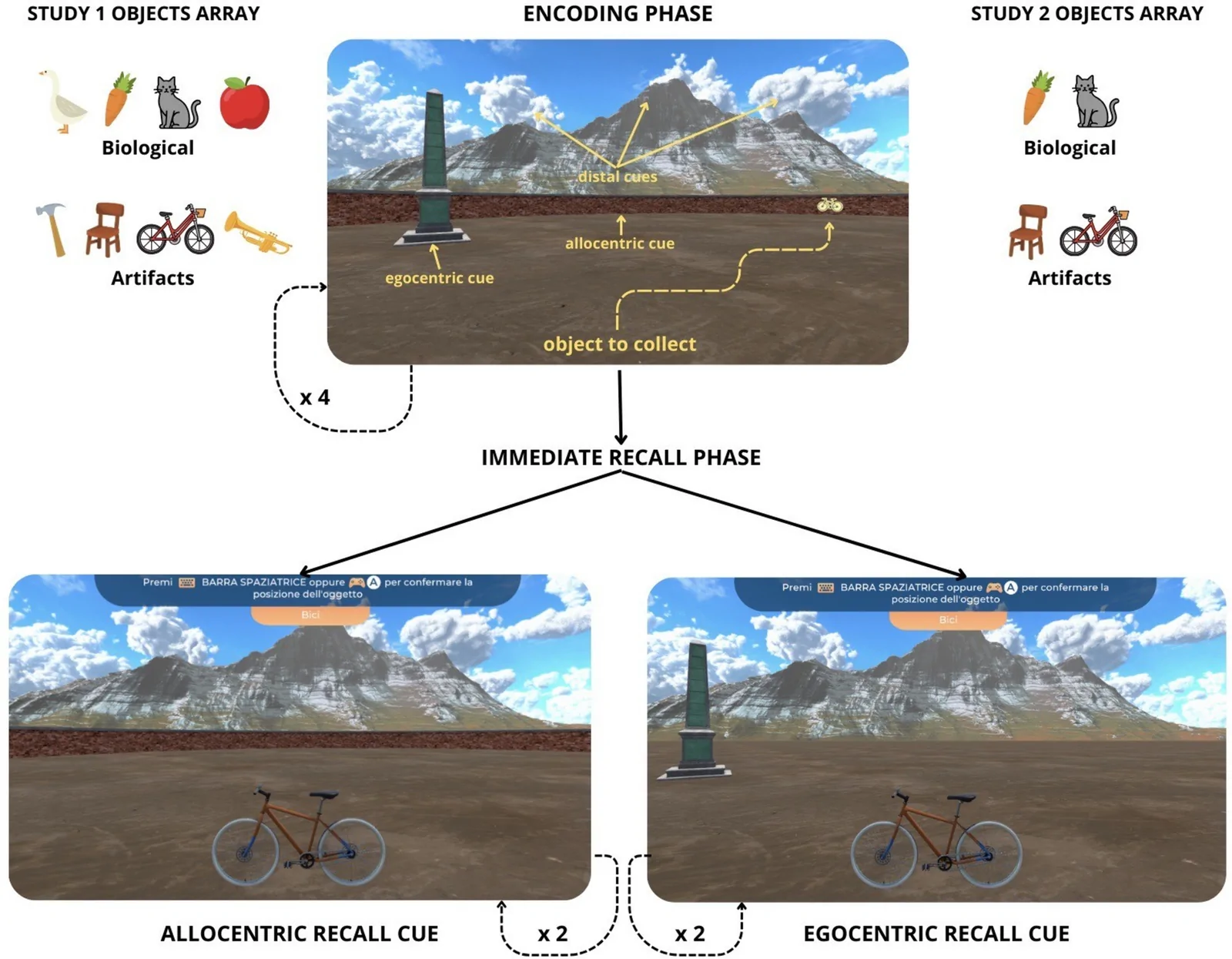
**Object-location spatial memory task**. Study 1: young adults; Study 2: older adults vs. amnestic mild cognitive impairment. During the encoding phase, participants were instructed to collect items one at a time and encode their locations within a circular arena (with a diameter of 50 virtual meters). Object locations could be learned using the arena’s boundaries (i.e., walls) and a fixed intra-arena landmark (i.e., obelisk). Items were presented randomly, and each object was collected four times in a random order. To reach the object, participants had to navigate to its exact location. Upon reaching it, the object disappeared, and they moved on to the next one. The starting position of this phase was always the center of the circular arena, facing an arbitrary North. During the immediate recall phase, participants had to remember and navigate to the exact location where the item (cued at the bottom of the screen) had previously been collected, then press the spacebar to respond. The next object was then presented. Either the wall or the obelisk was randomly removed, requiring participants to rely on allocentric (i.e., wall) or egocentric (i.e., obelisk) spatial memory recall cues. Fixed distal cues (e.g., mountain ranges, clouds) were available in all conditions to support orientation. Each object was tested twice under both recall conditions (32 total trials in Study 1 and 16 total trials in Study 2). (Color figure online)

Both studies employed a desktop-based virtual reality object-location memory task with landmark and boundary-based spatial recall (adapted from Guderian et al., 2015; Tuena et al., 2024). The task consisted of separate encoding and immediate recall phases. During encoding, participants navigated within a circular arena and collected objects, learning their locations with respect to both the arena boundary (i.e., walls) and a fixed intra-arena landmark (i.e., an obelisk). During recall, either the landmark or the boundary wall was randomly removed, requiring participants to rely on the other spatial cue to retrieve the object location. The dependent variable was the Euclidean distance between the encoding and recall locations (see Fig. 1 for a schematic overview of the task, and original publications for a detailed description).

The task design builds on dissociation between landmark-based and boundary-based spatial learning (Doeller et al., 2008). Evidence from both rodent and human studies suggest that learning goal locations relative to a proximal landmark is mediated by the dorsal striatum, whereas learning locations relative to environmental boundaries relies mainly on the hippocampal areas (Doeller et al., 2008; Iaria et al., 2003; Packard & McGaugh, 1992). Critically, as explained before, this dissociation aligns with the broader distinction between egocentric and allocentric spatial processing (Sheynikhovich et al., 2025). Accordingly, processing a spatial location relative to an intramaze landmark can be achieved egocentrically (e.g., “*the object is to the left of the landmark from my current position*”), without requiring an allocentric map of the environment. In contrast, encoding a location relative to a geometrically circular boundary requires spatial computations characteristic of the allocentric hippocampal system (Bicanski & Burgess, 2020).

Crucially for the present work, object stimuli in both original studies were equally distributed across biological and artifact categories, enabling balanced, category-based analyses without post hoc stimulus selection biases. For Study 1, the number of objects was eight (four biological items and four artifacts), whereas in Study 2, it was four (two biological items and two artifacts). The number of objects to be memorized was chosen based on normative short-term memory span data for the Italian population (Monaco et al., 2013). For YA (age range 18–30), eight objects were presented, exceeding their mean span (around six items) to ensure a supra-span spatial task. For OA and aMCI, four objects were used, consistent with prior studies (De Tollis et al., 2021; Monaco et al., 2013) and with the reduced memory span observed in these populations (around five items for OA and four for aMCI). This approach ensures that the task remains appropriately challenging across age and cognitive status.

### Procedure

In the original Study 1, participants were administered a sentence recognition task and an object-location spatial memory task in a counterbalanced order (in the current study, we used only spatial memory performance). In the original Study 2, the protocol was divided into two sessions. In the first session, participants were administered a comprehensive neuropsychological battery. In the second session, participants completed the object-location spatial memory task. Instructions provided to participants were the same for both studies (intentional encoding). For details on the neuropsychological battery administered to OA and aMCI, and on the experimental procedures, see the original publications (Tuena et al., 2025, 2026).

### Data analysis

Analyses were conducted in RStudio (Version 3.6.3) using linear mixed-effects models, which were tested with analysis of variance (ANOVA). For both studies, fixed effects were tested using Type III ANOVA Wald chi-square tests (*car* package). Wald chi-square tests provide an asymptotic test of fixed effects that does not rely on approximating denominator degrees of freedom and are therefore appropriate when using complex linear mixed-effects models. When significant interactions emerged, post hoc comparisons were performed using the Bonferroni correction for multiple comparisons. Study 1 employed a fully within-subjects 2 × 2 design with the factors *Recall Cue* (egocentric vs. allocentric) and *Category* (biological vs. artifacts). Fixed effects included the recall cue and the object category. Random effects comprised participant ID (random intercepts and random slopes) and object ID (random intercepts). Study 2 adopted a 2 × 2 × 2 mixed design, with *Recall Cue* (egocentric vs. allocentric) and *Category* (biological vs. artifacts) as within-subjects factors, and *Group* (OA vs. aMCI) as a between-subjects factor.

In both secondary analyses, three covariates were included in the models to control for potential confounds related to stimulus properties: (1) object distance from egocentric and allocentric cues at encoding, (2) item familiarity, and (3) item visual complexity. Object distance from cues at encoding was operationalized as the Euclidean distance of each object encoding location from the intra-arena landmark cue (obelisk; egocentric) and from the nearest point on the boundary circumference (wall; allocentric). Familiarity and visual complexity scores were derived from the ecological picture dataset validated in the Italian population by Viggiano and colleagues (2004). Effect sizes were reported as partial eta squared (η_p_^2^) and interpreted according to conventional benchmarks (small = 0.01, medium = 0.06, large = 0.14). The alpha level was set at.05 for all analyses. Outlier detection was performed using the interquartile range (IQR) method, applied separately for each experimental condition. In Study 1, this procedure excluded 46 observations (3.9% of the dataset). In Study 2, no outliers were identified. Sensitivity analyses were conducted to evaluate statistical power. Study 1 was adequately powered (≥ 80%) to detect large effects (*f* ≥ 0.50) but had limited power (33.7%) for the observed medium-large effect (*f* = 0.253). Study 2 was adequately powered for large effects (*f* ≥ 0.314) but had limited power for the observed effects: category main effect (*f* = 0.10, power = 14.6%), category-by-recall-cue interaction (*f* = 0.04, power = 6.5%), and the three-way interaction (*f* = 0.03, power = 6.0%).

## Results

A summary of the three groups is reported in Table 1. An extended neuropsychological table is reported in the original publication of Study 2. Crucially, OA and aMCI did not differ for computer experience (*p* = 0.27) and encoding time (*p* = 0.07).

**Table 1:** Participants characteristics

Variable	aMCI, N = 40	OA, N = 40	YA, n = 37	p-value
Age (years)	73.65 (6.27)	72.38 (6.23)	24.17 (2.51)	<.001†^,a,b^
Gender				.105^∫^
F	30 (75%)	23 (57%)	19 (53%)	
M	10 (25%)	17 (42%)	17 (47%)	
Education (years)	12.28 (4.49)	13.9 (3.37)	15.72 (2.31)	<.001†^,a,b^
MMSE (points)	27.48 (2.10)	28.52 (1.88)	-	.019^‡^
FAB (points)	14.99 (2.17)	17.05 (1.12)	-	<.001^‡^
CSS (points)	12 (7)	19 (7)	-	<.001^‡^
PM-I (points)	4.17 (2.27)	5.82 (1.28)	-	<.001^‡^
PM-D (points)	4.11 (2.22)	5.84 (1.19)	-	<.001^‡^
GDS (points)	2.65 (2.62)	1.93 (2.12)	-	.2^‡^
ADL (points)	5.95 (0.22)	5.92 (0.27)	-	.7^‡^
IADL (points)	7.93 (0.47)	7.93 (0.27)	-	.3‡

*aMCI* amnestic mild cognitive impairment, *OA* cognitively unimpaired older adults, *YA* young adults, *F* female, *M* males, *MMSE* mini-mental state examination, *FAB* frontal assessment battery, *CSS* Corsi supra-span learning, *PM* prose memory immediate (I) and delayed (D), *GDS* geriatric depression scale-short, *ADL* activities of daily living, *IADL* instrumental activities of daily living. Mean and standard deviation are reported for continuous variables. Neuropsychological tests were not administered to YA./pp‡ Welch two sample test, † F-test ANOVA, ∫ chi-squared. *a* post-hoc significant difference OA vs. YA, *b* post-hoc significant difference aMCI vs. YA/p

### Study 1

A linear mixed-effects model was fitted to examine the effects of object category (biological vs. artifacts) and recall cue type (allocentric vs. egocentric) on spatial memory recall errors. The model included baseline object distance from cues, object familiarity, and object visual complexity as covariates, with random intercepts for participants and objects, and random slopes for landmark type by participant.

Type III Wald chi-square tests revealed significant main effects for Category (χ^2^ = 13.53, *p* <.001, η_p_^2^ = 0.38) and Recall Cue (χ^2^ = 13.06, *p* <.001, η_p_^2^ <.001). Critically, these main effects were qualified by a significant Category × Recall Cue interaction (χ^2^ = 22.08, *p* <.001, η_p_^2^ =.06). Among covariates, baseline distance significantly predicted error scores (χ^2^ = 5.67, *p* =.017, η_p_^2^ =.05), while familiarity (χ^2^ = 1.38, *p* =.241, η_p_^2^ =.27) and complexity (χ^2^ = 3.00, *p* =.083, η_p_^2^ =.47) were nonsignificant.

**Fig. 2:**
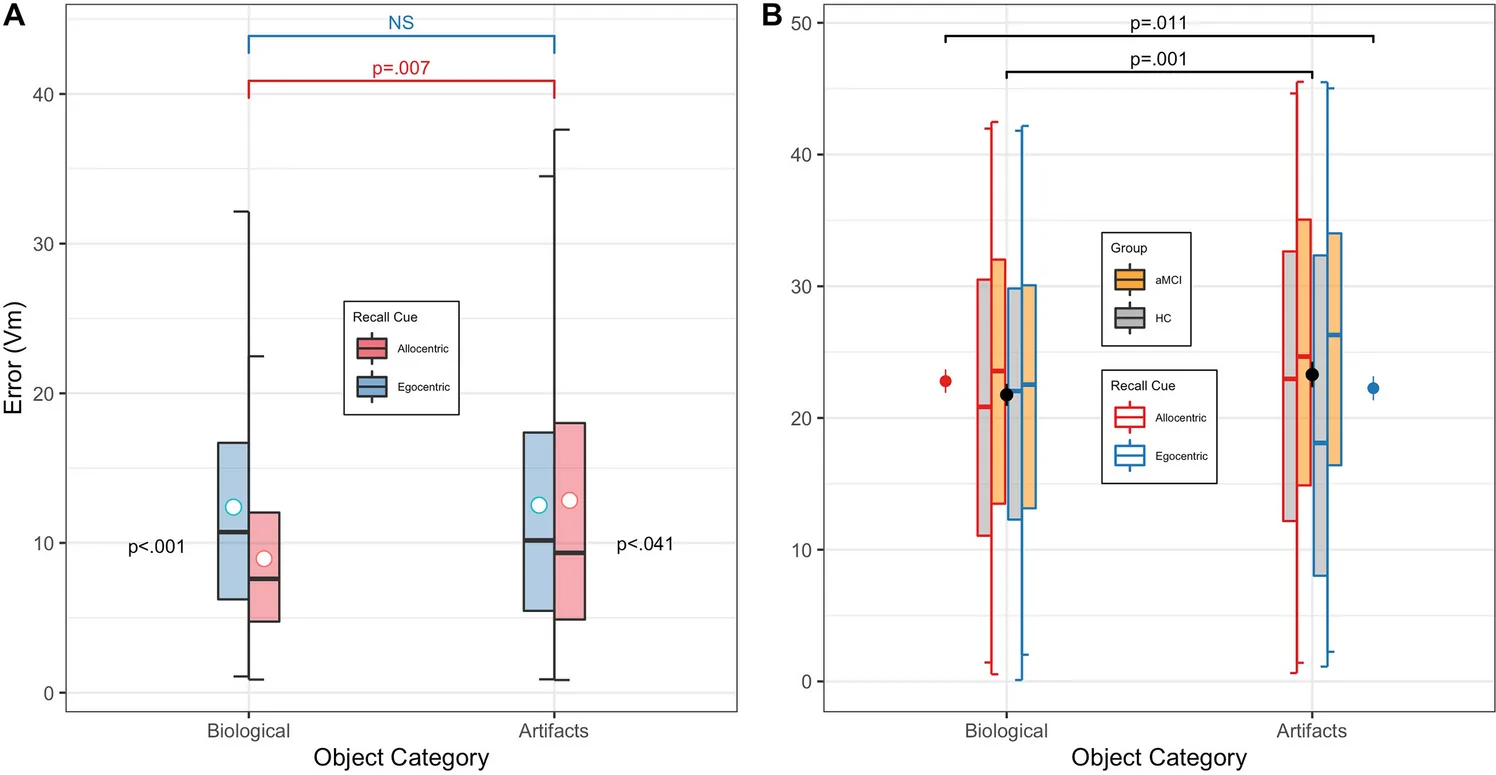
**A**) Significant category by recall cue interaction in young adults. Box plots show the median and IQR, whiskers show data range (IQR × 1.5), and dots show the mean for each condition. P-values were computed using estimated marginal means; comparisons therefore refer to means, not to the medians. **B**) Biological advantage in spatialmemory performance in aging. Biological advantage in spatial memory performance in aging. Box plots show the median and IQR, and whiskers show the data range (IQR × 1.5). Black dots show the mean and 95% CI and represent the main effect of object Category on spatial memory error across groups and recall cues; red (allocentric) and blue (egocentric) dots show the mean and 95% CI and represent the main effect of Recall Cue on spatial memory error across groups and categories. The main effect of the group variable and the two-way and three-way interactions were not significant VM = virtual meters; NS = not significant; OA = older adults; aMCI = amnestic mild cognitive impairment. (Colorfigure online)

Post hoc analyses with the Bonferroni correction revealed that the interaction showed different patterns across categories. Among biological items, participants showed significantly greater errors with egocentric than with allocentric recall cues (est. diff. =  − 2.45, *SE* = 0.69, *t* =  − 3.54, *p* <.001). Conversely, within artifacts, errors were significantly higher with the allocentric than the egocentric recall cue (est. diff. = 2.64, *SE* = 1.29, *t* = 2.05, *p* =.041). Between-category comparisons showed that artifacts produced significantly higher errors than biological items in the allocentric cue condition (est. diff. =  − 3.74, *SE* = 1.03, *t* =  − 3.63, *p* =.007), while no significant difference emerged in the egocentric cue condition (est. diff. = 1.35, *SE* = 0.97, *t* = 1.39, *p* =.211). Figure 2A shows this result.

### Study 2

A linear mixed-effects model was fitted to examine the effects of object category (biological vs. artifacts), recall cue type (allocentric vs. egocentric), and group (OA vs. aMCI) on spatial memory errors. The model included baseline object distance from cues, object familiarity, and object visual complexity as covariates, with random participant intercepts. An initial model with random intercepts for both participants and objects failed to converge due to overparameterization, necessitating the removal of the object random effect and the participant random slope.

Type III Wald chi-square tests revealed significant main effects for Category (χ^2^ = 10.81, *p* =.001, η_p_^2^ =.010) and Recall Cue (χ^2^ = 6.41, *p* =.011, η_p_^2^ =.010). Descriptive statistics showed that artifacts were recalled farther from the actual encoding location than biological items (artifacts: *M* = 23.3, *SD* = 12.5; biological: *M* = 21.8, *SD* = 10.8; see Fig. 2B). Between Recall Cue conditions, the allocentric recall cue yielded slightly greater distances than the egocentric cue (allocentric: *M* = 22.8, *SD* = 11.6; egocentric: *M* = 22.3, *SD* = 11.8 see Fig. 2 B). The main effect of Group was non-significant (χ^2^ = 1.46, *p* =.228, η^2^_p_ =.09). Critically, all two-way interactions were non-significant: Category by Recall Cue (χ^2^ = 0.05, *p* =.829, η_p_^2^ =.002), Category × Group (χ^2^ = 0.14, *p* =.703, η_p_^2^ =.002), and Recall Cue × Group (χ^2^ = 0.06, *p* =.804, η_p_^2^ =.002). The three-way interaction was also nonsignificant (χ^2^ = 1.27, *p* =.260, η_p_^2^ =.001). Among covariates, baseline distance was a significant predictor (χ^2^ = 11.10, *p* <.001, η_p_^2^ =.009), while familiarity (χ^2^ = 2.46, *p* =.117, η_p_^2^ =.002) and complexity (χ^2^ = 0.92, *p* =.336, η_p_^2^ =.001) were non-significant. Importantly, in the original analysis of Study 2, recall cue performance between groups was not affected by age, education, gender, retrieval time, or global cognition (see original publication section, differences between aMCI and HC and spatial memory recall cues).

## Discussion

The present work investigated whether the biological relevance of objects modulates spatial memory performance depending on the reference frame available at retrieval. In Study 1, young adults (YA) showed a significant interaction between object category and recall cue: biological entities were recalled more accurately under allocentric than egocentric retrieval, while artifacts showed the opposite pattern. However, the category difference was significant only under allocentric retrieval. In Study 2, which included older adults (OA) and patients with amnestic mild cognitive impairment (aMCI), the biological advantage was preserved: biological items were spatially located more accurately than artifacts, regardless of the retrieval condition; however, we did not observe an interaction between object category and recall cue.

The first implication of these findings concerns the nature of spatial representations. While research has traditionally focused on the role of environmental factors (Burgess, 2006; Wang & Spelke, 2000), as well as on task demands and the scale of the environment (Iachini, 2024; Richardson et al., 1999), in shaping the use of egocentric and allocentric reference frames, our results suggest that the nature of *what* is being located may also play a role. This is broadly consistent with the relational memory framework, which proposes that the hippocampus binds object identity and spatial context through converging but partially distinct medial temporal lobe pathways (Eichenbaum & Cohen, 2014; Eichenbaum et al., 1999). Our findings might suggest that these pathways not only carry different types of information in parallel but that the properties of the object may influence how effectively its location is retrieved across different spatial reference frames. The pattern observed in Study 1 was consistent with predictions derived from the adaptive memory framework (Nairne, 2014; Nairne & Pandeirada, 2016), though the partial dissociation requires some consideration. The object category difference emerged specifically during allocentric retrieval, where biological entities were recalled more accurately than artifacts, whereas no such difference was observed during egocentric retrieval. One possibility is that allocentric processing, which relies on hippocampal spatial computations, is more sensitive to stimulus properties, whereas egocentric processing may support spatial recall comparably across object categories. The results of Study 2 are consistent with this interpretation: in OA and patients with aMCI, where allocentric processing is known to be compromised (Colombo et al., 2017; Ladyka‐Wojcik & Barense, 2021; Serino et al., 2014, 2025; Tuena et al., 2021), and object-location memory is particularly vulnerable (Castegnaro et al., 2022), the interaction between category and recall cue was no longer observed, while the biological advantage in overall spatial performance was preserved. This pattern suggests that the biological relevance of objects may influence spatial memory through partially distinct routes. In young adults, the allocentric advantage for biological items may depend more strongly on hippocampal processing and may therefore be more vulnerable to age-related decline. The biological advantage, however, may be sustained by processes that are at least partially independent of the spatial reference frame, such as the greater attentional capture elicited by survival-relevant stimuli (New et al., 2007), which may enhance spatial performance regardless of the retrieval condition.

However, as reported, the sensitivity analysis indicated that Study 2 had limited power to detect interaction: while the study was adequately powered to detect large effects, the power for the category-by-recall-cue interaction was 6.5%, and for the three-way interaction 6.0%. Therefore, the absence of these interactions must be interpreted cautiously. Although the Study 2 pattern is compatible with a reduced allocentric advantage for biological items in aging, the available data does not allow a strong conclusion on this point. Disentangling these possibilities will require future studies with larger, prospectively designed samples.

An important aspect of our study is adopting a broader distinction between biological entities and artifacts, rather than the animate-inanimate dichotomy. This choice was motivated by the adaptive memory framework, which emphasizes the relevance of survival-related information beyond animacy in the narrow sense (Nairne & Pandeirada, 2010). Notably, in the spatial domain, van Buren and Scholl (2017) demonstrated that even the perceived animacy of simple geometric shapes, driven purely by motion cues, enhances spatial memory performance. Whether a stricter animacy classification would differentially modulate the use of spatial reference frames (as we observed here for the biological category) remains an open question for future research.

Some limitations should be acknowledged. First, the present analyses were conducted on previously collected datasets. The original studies were designed to investigate spatial reference frame processing and were not specifically powered for category-level effects. Second, we controlled for item familiarity and visual complexity, as these were the only stimulus properties for which normative values were available for our objects in the Italian validation dataset (Viggiano et al., 2004). Other properties known to modulate the animacy advantage, such as emotional valence (Meinhardt et al., 2018), were therefore not included as covariates and should be considered in future studies using ad hoc stimulus sets. Third, the number of stimuli differed between the two studies (eight objects in Study 1 vs. four in Study 2) because the task difficulty was adapted to the memory capacity of different populations. While this choice is methodologically motivated, it may limit the comparability between the two studies.

## Conclusion

The present study provides initial evidence that the biological relevance of objects modulates spatial memory performance as a function of the reference frame used at retrieval, highlighting an overlooked interaction between object category and representational format in spatial memory. In young adults, biological entities benefited from allocentric retrieval, while artifacts benefited from egocentric retrieval. In aging, the biological advantage persisted, but the interaction with reference frame was no longer observed, a pattern that may reflect reduced allocentric contributions. These findings suggest that spatial memory is also shaped by the nature of what we need to remember: this factor has been largely overlooked in spatial cognition research, and it may have implications for the design of clinical assessment tools (Serino et al., 2025).

## Data Availability

The datasets analyzed in the present study were collected as part of two previously published studies. Data from Study 1 are publicly available on the Open Science Framework (https://osf.io/yvhra/). Data from Study 2 are available via Zenodo (https://zenodo.org/records/15119995) upon reasonable request. The study was not preregistered.

## Code availability

Code is available from the corresponding author upon reasonable request.

## Declarations

### Use of artificial intelligence

Claude (Anthropic) was used to revise selected parts of the manuscript for English-language clarity and word choice. The authors reviewed and edited all AI-assisted text and take full responsibility for the final content.
